# Enhancing the Sense of Attention from an Assistance Mobile Robot by Improving Eye-Gaze Contact from Its Iconic Face Displayed on a Flat Screen

**DOI:** 10.3390/s22114282

**Published:** 2022-06-04

**Authors:** Elena Rubies, Jordi Palacín, Eduard Clotet

**Affiliations:** Robotics Laboratory, Universitat de Lleida, Jaume II, 69, 25001 Lleida, Spain; helenarubies@gmail.com (E.R.); eduard.clotet@udl.cat (E.C.)

**Keywords:** sense of attention, eye-gaze, iconic face, big eyes, assistance robot

## Abstract

One direct way to express the sense of attention in a human interaction is through the gaze. This paper presents the enhancement of the sense of attention from the face of a human-sized mobile robot during an interaction. This mobile robot was designed as an assistance mobile robot and uses a flat screen at the top of the robot to display an iconic (simplified) face with big round eyes and a single line as a mouth. The implementation of eye-gaze contact from this iconic face is a problem because of the difficulty of simulating real 3D spherical eyes in a 2D image considering the perspective of the person interacting with the mobile robot. The perception of eye-gaze contact has been improved by manually calibrating the gaze of the robot relative to the location of the face of the person interacting with the robot. The sense of attention has been further enhanced by implementing cyclic face explorations with saccades in the gaze and by performing blinking and small movements of the mouth.

## 1. Introduction

Eye contact is a valuable communicative signal that allows for the extraction of socially relevant information such as state, behavior, intention and emotions [1]. The human face nonverbally expresses characteristics such as character, emotion and identity [2].

According to Yoshikawa et al. [3], the impression a person forms during an interaction is influenced by the feeling of being looked at, which depends on the eye-gaze response from the interlocutor. Yoshikawa et al. [3] demonstrated that a robot with responsive gaze also provides a strong feeling of being looked at. Similarly, as in a human interaction, mutual gaze also engages human–robot interaction [4,5,6,7,8], influences human decision making [9,10] and plays a central role in directing attention during communication [3].

There are many alternative ways to enhance the sense of attention from a robot. Barnes et al. [11] concluded that users prefer robots that resemble animals or humans over robots that represent imaginary creatures or do not resemble a creature, regardless of the type of interaction with the robot. Mutlu et al. [12] evaluated the effect of the gaze of a storytelling robot, concluding that participants preferred a robot looking at them during the storytelling. Mutlu et al. [13,14] also studied gaze mechanisms in multi-party human–robot conversations, concluding that the gaze allowed the robot to assign and manage the participant roles. Shintani et al. [15] analyzed role-based gaze conversational behaviors and developed a robot with human-like eye movements, obtaining smoother, more natural and more engaged human–robot interactions. Fukayama et al. [16] measured the impressions of users interacting with a robot in different social communicating scenarios and concluded that there is a correlation between the impression and the amount of gaze, the mean duration of the gaze and the gaze points. Lee et al. [17] designed a robotic gaze behavior based on social cueing for users performing quiz sessions in order to overcome in-attentional blindness, with the conclusion that the robotic gaze can improve the quiz scores when participants successfully recognize the gaze-based cues performed by the robot.

Similarly, Ghiglino et al. [18] verified that endowing artificial agents with human-like eye movements increased attentional engagement and anthropomorphic attribution. The conclusion was that users needed less effort to process and interpret the behavior of an artificial agent when it was human-like, facilitating human–robot interaction. Cid et al. [19] studied the mechanisms of perception and imitation of human expressions and emotions with a humanoid robotic head designed for human–robot interaction. The use of a robotic head allows for the interaction through speech, facial expressions and body language. Cid et al. [19] also presented a software architecture that detects, recognizes, classifies and generates facial expressions using the Facial Action Coding System (FACS) [20,21] and also compared the scientific literature describing the implementation of different robotic heads according to their appearance, sensors used, degrees of freedom (DOF) and the use of the FACS.

The new contribution of this paper is a proposal to enhance the sense of attention from an assistance mobile robot by improving eye-gaze contact from the face of the robot. This proposal is inspired by the contributions of Velichkovsky et al. [22] and Belkaid et al. [10]. Velichkovsky et al. [22] analyzed the implementation of different social gaze behaviors in a robot in order to generate the impression that a companion robot is a conscious creature. Velichkovsky et al. [22] evaluated the impression of the gaze behavior on humans in three situations: a robot telling a story, a person telling a story to the robot, and both parties solving a puzzle while talking about objects in the real world. The gaze behavior implemented in the robot consisted of alternating the gaze between the human, the environment and the object of the problem. The conclusion was that social gaze simulated by robots can make the human assign cognitive and emotional properties to the robot. Alternatively, Belkaid et al. [10] analyzed the effect of mutual gaze and adverted gaze between a robot and a human before making a decision. Belkaid et al. [10] analyzed a mechatronic (mechanistic mannequin-like) head with big spherical eyes with the conclusion that “robot gaze acts as a strong social signal for humans, modulating response times, decision threshold, neural synchronization, as well as choice strategies and sensitivity to outcomes”. Following these conclusions, the basic gaze implemented in an assistance mobile robot prototype has been revised in order to enhance the sense of attention from its iconic face displayed on a flat screen.

Human ocular motion has been deeply analyzed from an anatomical and physiological point of view; however, thus far, the development of robotic eyes has mainly focused on biomimetic mechatronic implementation [23,24,25] and on movement [19,25,26,27,28] rather than on the impression originated by the gaze implemented. The evaluation of the gaze is an open problem, and there is no quantitative method generally proposed to evaluate eye-gaze contact because it is based on subjective human perceptions that are generally influenced by pathologies such as strabismus. For example, eye-trackers are considered valid to estimate the location of the fixation point over a plain screen because they usually interpolate this location from a reduced set of initial eye-gaze calibrations performed at a specific distance [29]. In the case of using a screen to represent the face of the robot, an additional problem is the difficulty of simulating the effect of spherical eyes in a plain image considering the perspective of the person interacting with the mobile robot. Because of these difficulties, the gaze of a robot is usually mainly implemented only to provide a basic impression of a responsive robot [19,25], although there are other implementations such as, for example, the reduction of vision instability by means of the reproduction of the vestibulo–ocular reflex [27,28].

This paper proposes enhancing the sense of attention perceived from the iconic face displayed on the screen of an assistance mobile robot during an interaction. In this paper, the sense of attention has been interpreted as the perception of being looked at by the responsive eyes of the iconic face of the robot, which are displayed on a flat screen. This implementation has been validated with five people who work regularly with robots.

The gaze originally implemented in the assistance mobile robot used in this paper had seven predefined gaze orientations: forward, up, down, half left, half right, left, and right, in all cases with parallel eyes fixed on infinity. The use of these fixed predefined gaze orientations provided the impression of a responsive robot but was not able to generate a sense of attention. As described before, there are no tools to evaluate eye-gaze contact; thus, the perception of eye-gaze contact from the robot has been maximized by manually obtaining 169 eye-gaze calibration points relative to the location of the face of the person interacting with the robot. These calibration results are fully provided for additional evaluation and validation. Finally, the sense of attention has been further enhanced by implementing cyclic face explorations with saccades in the gaze and by performing blinking and small movements of the mouth.

## 2. Background

### 2.1. Simplified Geometric Definition of the Binocular Vision

**Figure 1:**
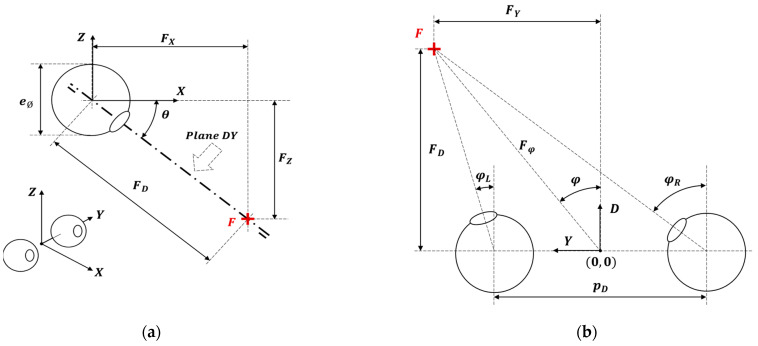
Simplified geometric interpretation of the eyes looking at a fixation point, F: (**a**) side view of the eye model; (**b**) representation of the plane of sight (Plane DY).

Binocular vision is a type of vision characterized by the use of two eyes capable of facing the same direction. Figure 1 shows a schematic geometric representation of the human eye model based on the simplified model proposed by Turski [30] in the case of the two eyes looking at a fixation point, F. Table 1 presents the notation of the geometric parameters described in Figure 1. Figure 1a presents the coordinate system (X,Y,Z), whose center is located between the two eyes and is aligned with the center of the eyeballs. Figure 1a also shows a side view of the eyes looking down at a fixation point F at ($F_{X},F_{Y},F_{Z}$). The sight plane DY is represented laterally, with a deviation angle θ referred to the horizontal XY plane. The main geometric parameters represented are the eyeball diameter ($e_{Ø}$), the angular deviation of the sight plane (θ), the coordinates of the fixation point in the main coordinate system ($F_{X},F_{Z}$) and in the sight plane ($F_{D}$). Figure 1b is a top view of the sight plane (Plane DY), which contains the fixation point F. The angular orientation that the left and right eyes take to look at the fixation point is defined by the angles $\varphi_{L}$ and $\varphi_{R}$, respectively. The main geometric parameters represented are the coordinates of the fixation point in the sight plane ($F_{D},F_{Y}$), the pupillary distance ($p_{D}$) and the angular deviation of the eyes to the fixation point ($\varphi_{L},\varphi_{R}$).

The sight angle referring to the XY plane and the angular orientation of the eyes to the fixation point in this simplified representation are computed using:

(1) $$\theta =atan\left(\frac{F_{Z}}{F_{X}}\right)$$

(2) $$F_{D}=\sqrt{F_{X}^{2}+F_{Z}^{2}}$$

(3) $$F_{\varphi }=\sqrt{F_{Y}^{2}+F_{D}^{2}}$$

(4) $$\varphi_{L}=atan\left(\frac{F_{Y}-p_{D}/2}{F_{D}}\right)$$

(5) $$\varphi_{R}=atan\left(\frac{F_{Y}+p_{D}/2}{F_{D}}\right)$$

When the human eyes are looking forward (to the infinity), their angular orientation is $\varphi_{L}=\varphi_{R}=0{}^{\circ}$, and the sight angle is θ=0°. In the condition of stable eyes fixation (without movements), the horizontal visual field is around 210° (in which there are 120° of binocular vision), and the vertical visual field is around 150° [31,32]. Finally, anthropometric databases show a mean adult pupillary distance ($p_{D}$) of 63 mm [33].

### 2.2. Eye Movements

Eye movement refers to the voluntary or involuntary movement of the eyes during the acquisition of visual information [34] in order to fix the image from the fixation point in the fovea, which is the central area of the retina [35,36]. In the human eye, the fovea is the point with clearest vision, highest sensitivity to fine details and color [37] and highest visual acuity in the direction where the eye is pointed. However, the fovea receives information from a range of only two degrees of the visual field [38]; thus, the eyes have to be moved in order to acquire more visual information. The eye movements can be physiologically classified according to different criteria [34,39] in fixation eye movements, gaze-shifting movements, involuntary or reflex gaze-shifting movements and relative eye movements.

#### 2.2.1. Fixation Eye Movements

The fixation movements are small eye movements around a stationary fixation point [34,39] that are used to acquire more visual information with the fovea. The fixation movements are:

**Microsaccades**. Small and rapid eye movements around the fixation point.

**Ocular drifts**. Smooth and slow motion of the eye around the fixed object.

**Ocular microtremors**. Quick and synchronized oscillations of both eyes with high frequency and very small amplitude.

#### 2.2.2. Gaze-Shifting Eye Movements

The gaze-shifting movements are rapid and ballistic eye movements between different fixation points [39,40]. The gaze-shifting eye movements are:

**Saccades**. Rapid eye movements between different fixation points. Saccades are used to scan big areas with the fovea [41] moving the eyes at their maximum speed [42]. The total angular displacement of the eye performing a saccade is a few minutes of arc. This rapid eye movement is clearly perceived while performing eye-gaze contact during a short-distance social interaction.

**Smooth pursuit**. Tracking of a moving object with the eyes to keep its moving image projected on the fovea [39,42].

#### 2.2.3. Involuntary Gaze-Shifting Eye Movements

The involuntary or reflex gaze-shifting movements are rapid and ballistic eye movements between different fixation points [39]:

**Vestibulo-ocular reflex**. Reflex eye movement that stabilizes the gaze during head movements, compensating the motion of the head by turning the eyes in the opposite direction [39].

**Optokinetic response**. Reflex eye movement that returns the eyes to the first position at which a moving object was seen before going out of the vision field [39].

#### 2.2.4. Relative Eye Movements

The relative movements of the eyes can be also classified according to the number of eyes involved during gaze or according to their relative motion [34,39,43]:

**Duction**. Small movement of only one eye while the other remains static. For example, this movement can originate in the case of one eye with the fovea aligned with the fixation point.

**Version**. Small synchronous movement of the two eyes in the same direction [43]. For example, this movement can originate when the fixation point is moving from the right to the left.

**Vergence**. Small synchronous movement of the two eyes in opposite directions to focus the object of interest in the fovea of each eye and maintain single binocular vision [39,42,43]. For example, this movement can originate when the fixation point is radially approaching or receding.

### 2.3. Eye Movements When We Look at Faces

In human interactions, eye movements when we look at faces enable eye-gaze contact and provide non-verbal communication. Yarbus [34] developed a method for recording eye movements over long periods of time and studied how participants looked at the photo of a face. Results showed a cyclic fixation behavior when viewing the faces, cycling periodically through the triangle of the eyes, nose and mouth, and focusing mostly on these points. Blais et al. [44] later validated this cyclic fixation behavior when looking at faces, reporting that the cyclic sequence may be affected by the cultural background of the observer. Hsiao et al. [45] performed a face recognition study and reported gazing at fixation points with durations ranging from 235 to 340 ms. This paper explores the implementation of saccades in the gaze of the iconic face in order to imitate this cyclic fixation sequence when humans look at faces.

## 3. Materials and Methods

The materials used in this paper are an assistance mobile robot prototype and the onboard cameras of the mobile robot.

### 3.1. Assistant Personal Robot

**Figure 2:**
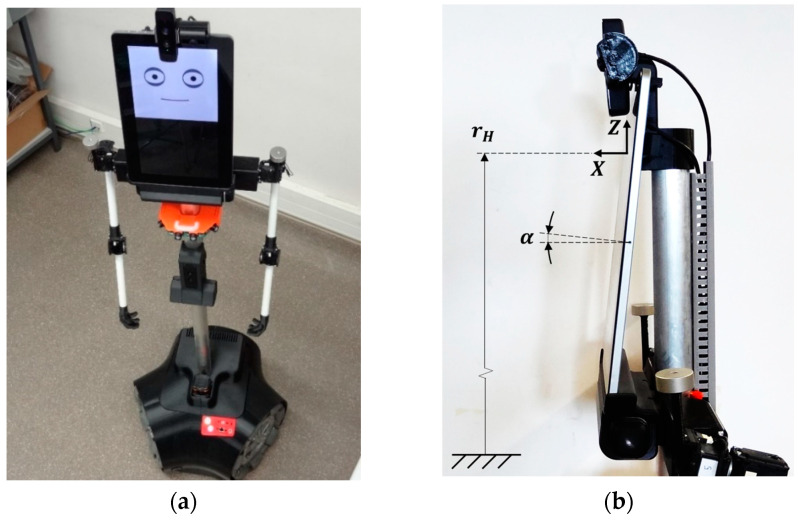
Image showing the assistance mobile robot used in this paper: (**a**) entire robot; (**b**) side-view detail of the screen used as a head, the coordinate system, the height of the eyes of the robot referred to the ground ($r_{H}$), and the inclination angle of the screen (α).

The mobile robot used in this paper is a prototype developed at the University of Lleida under the concept of Assistant Personal Robot (APR) [46] (1.76 m, 30 kg). Figure 2a shows an image of the prototype implementation used in this paper, the APR-02, which includes sensors and processing capabilities in order to operate autonomously as an assistance mobile robot. The mobile robot includes a flat capacitive touch-screen monitor (Geichic On-Lap 1303i) in the upper part used as a visual display unit and to provide touch feedback from users. This compact liquid crystal display (LCD) monitor has an aspect ratio of 16:9, a resolution of 1920 × 1080 pixels, a weight of 898 g and an angle of view of 178° with an average power consumption of 4.0 W (5.0 V, 0.8 A). The monitor has a micro-HDMI connector, a micro-USB connector for power supply, another micro-USB connector to provide the tactile feedback, and an audio jack for onboard speakers. The monitor is placed vertically on the mobile robot (Figure 2). It is connected to the onboard portable computer (PC) using the HDMI interface, and the USB interface provided is used to obtain the touch feedback from the screen. Figure 2 also shows the two cameras available above the monitor. The RGB-D camera is a Creative 3D Senz, weighing 271 g, with an average power consumption of 2.0 W (0.4 A at 5.0 V). This camera is placed vertically above the center of the monitor in order to have the highest field of view in the vertical plane of the mobile robot and to obtain complete face images of users of different heights interacting with the mobile robot. The second RGB camera (ELP-USBFHD01M-L180, power consumption 220 mA) has a panoramic lens and is located beside the RGB-D camera. The mobile robot additionally uses a LIDAR (Hokuyo UTM-30LX, 12 V and 1.0 A) for path planning, trajectory control and obstacle avoidance. The detailed evolution of the APR mobile robots is described in [46,47]. Currently, the APR-02 prototype is being used as a testbench for self-location [48], omnidirectional wheel evaluation [49], and trajectory and odometry evaluation [50,51]. Figure 2b shows the coordinate system defined by the center of the eyes (X,Y,Z), the height of the eyes of the robot ($r_{H}$) referred to the ground, and the inclination angle of the screen (α), which is 7°.

### 3.2. Iconic Face Implemented in the Assistance Mobile Robot

**Figure 3:**
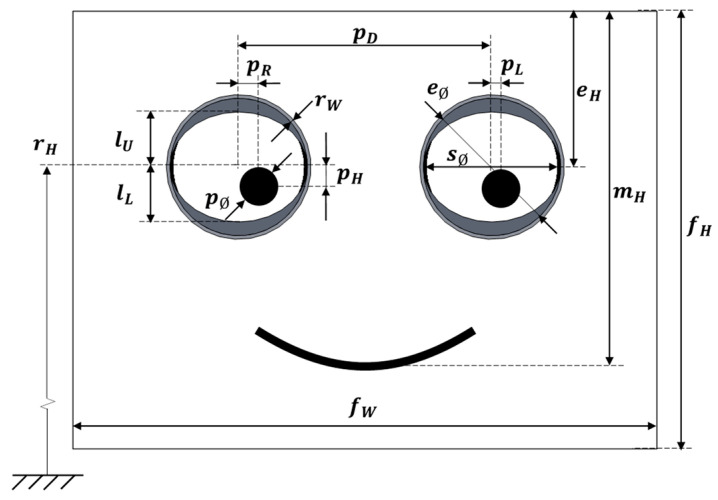
Image and parameters that define the iconic face implemented in the assistance mobile robot.

Figure 3 shows the detail of the iconic face implemented in the mobile robot, which was proposed and described in [47]. This iconic face has big eyes in order to enhance trustworthiness of the robot [52]. The iconic face establishes the eye-gaze contact with the person interacting with the mobile robot or located around the mobile robot. This face was implemented as an agent with configurable parameters such as the relative inner object location and size, the width of the lines and the colors used in the different schematic graphic objects represented in the iconic face.

The iconic face is displayed in the panoramic screen available in the upper part of the mobile robot (see Figure 2). The screen is oriented vertically, and the face is displayed in the half-upper part, with a facial width-to-height ratio (fWHR) higher than 1 [53]. The half-lower part of the screen is available to display additional information such as the identification of the mobile robot or the task in progress. Figure 3 shows the default iconic face that represents the mobile robot used in this paper [47] and its main parameters, and Table 2 summarizes the default values of the most representative parameters.

In this paper, the location of the pupils is computed and specified using a relative percentage scale in which 0% represents the location at the center of the eye and 100% the extreme position in which only half of the pupil is visible in the eyes. The relative horizontal percentage location of the pupil of the left eye ($D_{L}$) and of the right eye ($D_{R}$) and the common relative vertical percentage location of the pupils of both eyes (H) are computed using:

(6) $$D_{L}=\frac{100\cdot p_{L}}{s_{Ø}/2}$$

(7) $$D_{R}=\frac{100\cdot p_{R}}{s_{Ø}/2}$$

(8) $$H=\frac{100\cdot p_{H}}{s_{Ø}/2}$$

Similarly, the relative eyelid percentage scale of both eyes (upper, $C_{U}$ and lower, $C_{L}$) is computed using:

(9) $$C_{U}=\frac{100\cdot l_{U}}{e_{Ø}/2\;}$$

(10) $$C_{L}=\frac{100\cdot l_{L}}{e_{Ø}/2}$$

where $C_{U}=C_{L}=0\%$ represent the eyes closed (covered) and $C_{U}=C_{L}=100\%$ the eyes totally opened (uncovered).

Finally, the line of the mouth is computed from a percentage value (M) that directly modifies the amplitude of the smile: M=100% represents a high smiling degree, and M=0% represents the minimum smiling degree (mouth as a straight line).

### 3.3. Fontal Images Provided by the Two Cameras above the Monitor

**Figure 4:**
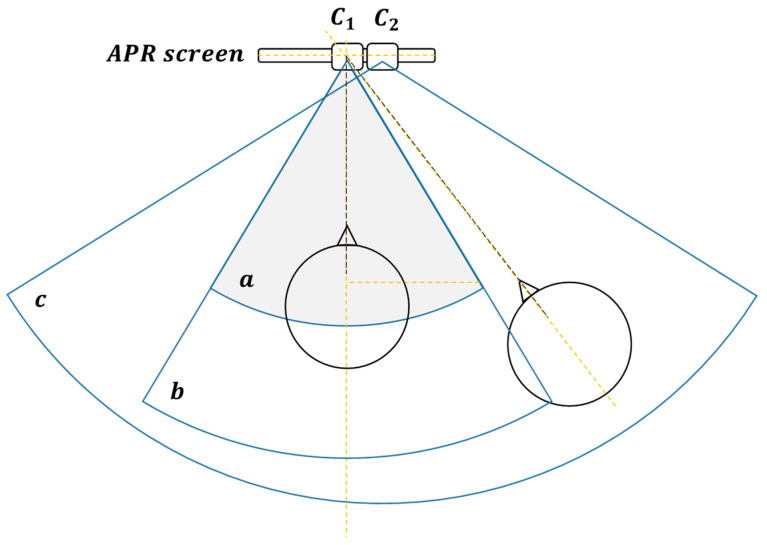
Approximate representation of the field of view of the frontal upper cameras of the APR-02 mobile robot: $C_{1}$ is the RGB-D camera, and $C_{2}$ the panoramic RGB camera.

Figure 4 shows a representation of the frontal field of view provided by the two cameras mounted above the monitor of the APR-02 mobile robot. The RGB-D camera is labeled as $C_{1}$ in Figure 4 and *a* and *b* represent its field of view, which in this paper is segmented as: *a*, zone for interaction and *b*, proximity zone. The RGB panoramic camera is labeled as $C_{2}$ in Figure 4, and *c* represents its wider field of view.

**Figure 5:**
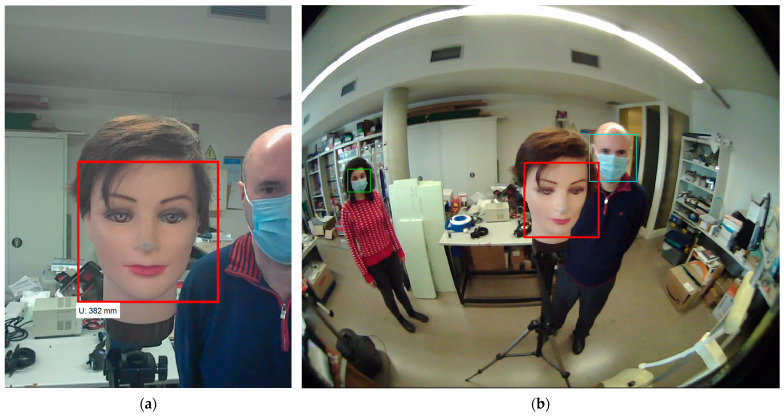
Figure representing two typical images provided simultaneously by (**a**) the upper frontal RGB-D camera (480 × 640 pixels); (**b**) the RGB panoramic camera of the APR-02 mobile robot (1280 × 1024 pixels). The image shows the mannequin face and two authors of this paper; the rectangles depict the faces detected in the images.

The RGB-D camera is accessed through a proprietary software development kit (SDK) that must be used to access, individually or collectively, the different streams of information provided by the camera. The RGB panoramic camera provides support to the standard USB video class (UVC) driver and is accessed as a conventional webcam. Figure 5 shows two example images representing the field of view provided by the two frontal cameras. These two RGB images have been acquired simultaneously from the upper frontal RBG-D camera and the RGB panoramic camera. The images show a mannequin head and two authors of this paper in front of the mobile robot, and the face masks were because of the COVID-19 public-health pandemic restrictions during the development of this paper. The faces available in the images are detected with the Viola and Jones algorithm [54] because it provides real-time performances in embedded systems with limited resources [55]. The faces detected in both images have been labeled with a rectangle and the central and closest face detected with the RGB-D camera has been identified with a red rectangle in both images.

The RGB-D camera is capable of providing RGB images of different resolutions: 1280 × 720, 640 × 360, 320 × 240 and 160 × 120, with 640 × 480 as the one used by default in the mobile robot (see Figure 5). The RGB panoramic camera is capable of providing RGB images of different resolutions: 1920 × 1080, 1280 × 1024, 1280 × 720, 1024 × 768, 800 × 600, 640 × 480 and 320 × 240, with 1280 × 1024 as the one used by default in the mobile robot (see Figure 5).

**Figure 6:**
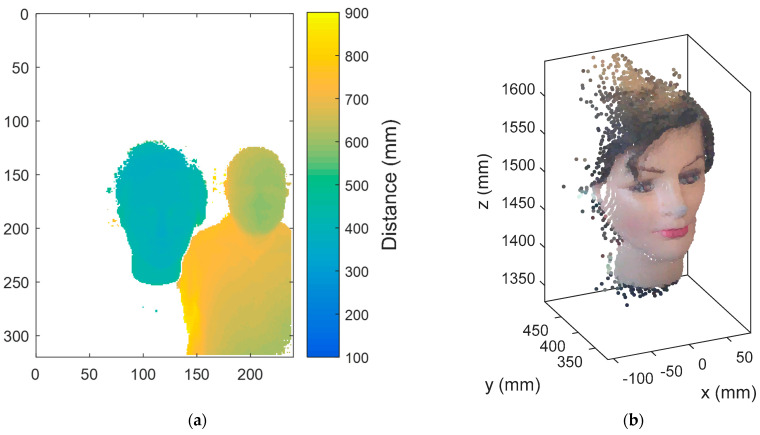
Figure showing (**a**) the representation of a typical depth image provided by the RGB-D camera (240 × 320 pixels); (**b**) the representation of the XYZC point cloud of the nearest face detected in the RGB image provided by the RGB-D camera (3233 data points). The XYZC point cloud has been analytically computed from the depth and RGB streams.

Figure 6a shows the depth image or stream provided from the RGB-D. The only resolution available for this depth image is 320 × 240 pixels, in which each pixel depicts a radial distance information, represented in the image as a color scale. This depth image stream allows for the implementation of simple distance segmentation algorithms. The RGB-D camera uses an infrared (IR) illumination and an IR camera to compute the depth information in a range from 0.5 to 1.5 m. The depth image of Figure 6a has been obtained simultaneously with the RGB image shown in Figure 5a. The combination of these two images can be used to deduce a point cloud data representation in which each point is identified by its (x, y, z) coordinates and by the color of the point detected; this point cloud data is labeled as XYZC in this paper. Unfortunately, the RGB-D camera used in this paper does not compute internally the XYZC point cloud; thus, it must be computed externally using the SDK libraries.

Figure 6b shows the XYZC point cloud data corresponding to the distance-segmented nearest face detected in front of the RGB-D camera. This face has been detected at an average nose distance of 382 mm. The XYZC point cloud of the face has been obtained by combining segmented distance information and color information, described by a total of 3233 points. In general, the XYZC point cloud is limited by the resolution of the depth image; thus, it is not useful the use of high resolution in the RGB images acquired by the RGB-D camera as they require more processing to compute the XYZC point cloud without providing any improvement. The RGB-D camera can also be used to recognize emotions [56] and to imitate human head movements [57].

### 3.4. Measurement Setup

**Figure 7:**
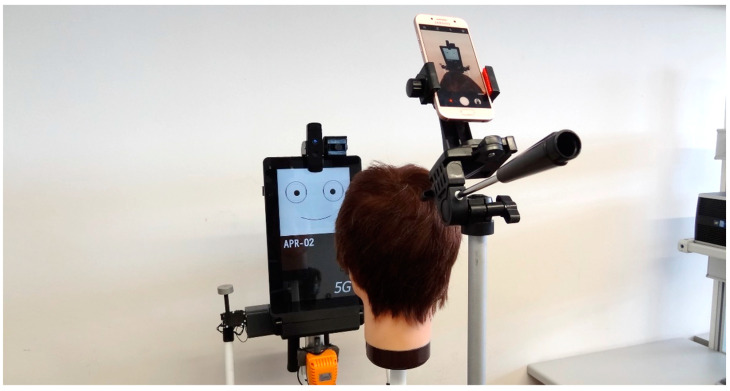
Image of the measurement setup showing the assistance mobile robot, the mannequin head and the camera used to take pictures of the robotic eye-gaze response.

Figure 7 shows the measurement setup used in this paper to enhance the sense of attention from the assistance mobile robot. The measurement setup is composed of the mobile robot APR-02, a human-scale mannequin head and a smartphone used as a camera to take pictures of the robot eye-gaze response from different points of view. The use of a mannequin head allows for the development of large experimentation rounds and the exact placement of the face/head in front of the mobile robot in different experiments.

## 4. Imitation of a Human Gaze Looking at a Face

This section describes the procedures proposed to imitate human gaze during a social interaction. The look-at-face gaze has been implemented by: locating the eyes and mouth of the person in front of the mobile robot, controlling the gaze of the iconic face, and by simulating a cyclic exploration of the face of the person in front of the mobile robot.

### 4.1. Holistic Location of the Eyes and Mouth of the Person in Front of the Mobile Robot

The enhancement of the sense of attention from an assistance mobile robot requires a precise control of the eye-gaze contact with the person interacting with the mobile robot, and this requires an accurate detection of the face, eyes and mouth. As described in Section 3.3, the procedure used to detect a person/face in front of the mobile robot is based on the face detection algorithm proposed by Viola and Jones [54] that identifies faces in images and returns a square at the locations of the faces. The combined use of this face detection algorithm and the distance information provided from the RGB-D camera allows for a precise detection and a precise spatial location of the faces of the people standing in front of the mobile robot. However, this precise distance localization is limited by the field of view of the RGB-D camera (see Figure 5).

The Viola–Jones algorithm [54] was proposed to detect faces in images. This classification procedure can also be applied to directly detect a variety of object classes such as eyes, mouths or noses by training the cascade detection of simple features [58,59]. Nevertheless, these specific detections usually require more computational resources. Originally, the Viola–Jones algorithm [54] was tailored to detect unmasked frontal upright faces, but it is now able to detect slightly turned faces, slightly rotated faces, frontal faces wearing surgical masks hiding the mouth, and slightly turned or rotated masked faces (see Figure 5). These good results with masked faces are because the main features detected by the algorithm are the eyes and the eyebrows.

**Figure 8:**
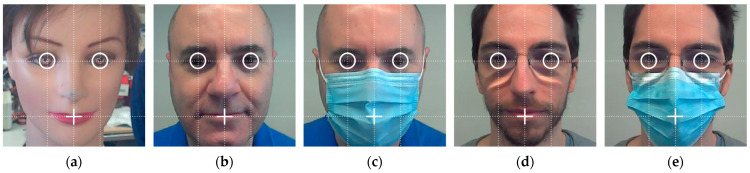
Representation of the face square areas identified by the Viola–Jones algorithm [54] and representation of the average fixed proportions holistically proposed to locate the eyes and mouth in the cases of: (**a**) human-sized mannequin; (**b**) user 1; (**c**) user 1 masked; (**d**) user 2; (**e**) user 2 masked.

Although facial proportions, angles, and contours vary with age, sex, and race [60], this paper applies a holistic approach to detect the relative location of the eyes and mouth in the square area of the image identified as a face by the Viola–Jones algorithm [54]. This holistic approach is based on averaging the location of the eyes and mouth in the face-area detected by the Viola–Jones algorithm [54]. This holistic approach has the advantage of not requiring additional computational resources. Figure 8 shows the face square image sections detected in the case of the mannequin face used in this paper and in the case of masked and unmasked faces of two authors of this paper. Figure 8 also shows the application of the holistic location of the eyes and mouth. The lines define the common holistic average proportions, a circle localizes the eyes, and a cross localizes the mouth.

**Figure 9:**
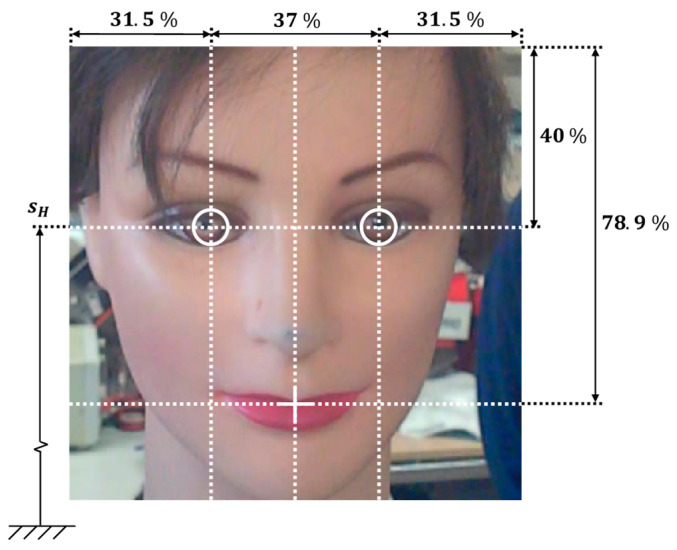
Holistic face proportions proposed in this paper to detect the eyes and mouth in a square image section classified as a face by the Viola–Jones algorithm [54]. The height of the sight plane of the face detected is labelled as $s_{H}$.

Figure 9 summarizes the average holistic proportions obtained. They are used to roughly locate the eyes and mouth of a person in front of the mobile robot relative to the size of the face detected. The average holistic proportions are: a pupillary distance of 37%, eyes height of 40%, and a mouth height of 78.9% of the size of the face detected. The localization of the eyes and mouth of the person in front of the mobile robot allows for a precise implementation of the cyclic exploration of the face in order to enhance the sense of attention from the assistance mobile robot.

### 4.2. Control of the Gaze of the Iconic Face Looking at a Human Face

This section presents the experimental procedures implemented to determine the gaze of the iconic face of the mobile robot looking at a human face. The control problem consists of the determination of the position of the pupils in the eyes of the iconic face ($D_{L}$, $D_{R}$ and H values) in order to focus the sight on a fixation point (F). In all the experiments conducted in this paper, a mannequin head was placed at different distances and orientations from the mobile robot, and the position of the pupils in both eyes of the iconic face was manually adjusted until the perception of eye-gaze contact (from the mobile robot to the mannequin head) was maximized.

In this section, the position of the fixation point ($F_{X},F_{Y},F_{Z}$) that defines the location of the face interacting with the robot is between its eyes and in the face plane (see Figure 9). The use of a mannequin head in the experimental setup is determinant because a fixed and static face in front of the mobile robot ensures the replicability of the experiments. During the experiments, the fixation point that defines the location of the face was measured manually in order to achieve the best precision, but the mobile robot is prepared to automatically to obtain this location from the frontal RGB-D camera and the Viola–Jones [54] algorithm.

#### 4.2.1. Determination of a Short-Distance Look-at-Face Gaze from the Iconic Face

The procedure proposed to develop the look-at-face gaze and implement eye-gaze contact from the iconic face of the mobile robot during a short-distance interaction (x ≤ 0.95 m) is based on the calibration of $D_{L}$, $D_{R}$ and H for fixation points placed at different ($F_{X},F_{Y},F_{z}$) positions. Table A1 shows the short-range calibration data of $D_{L}$ and Table A2 of $D_{R}$ for different ($F_{X},F_{Y}$) values with $F_{Z}=0$ in order to avoid the influence of a vertical deviation in the look-at-face gaze. Table A3 shows the short-range calibration data of H for different ($F_{X},F_{Z}$) in the case of a face centered in front of the mobile robot ($F_{Y}=0$) in order to avoid the influence of a lateral deviation in the look-at-face gaze.

**Figure 10:**
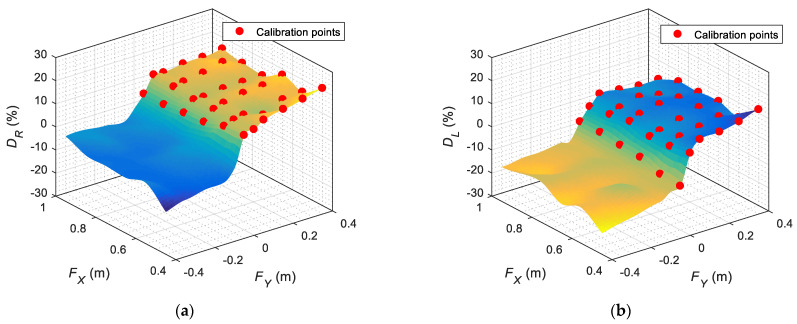
Representation of the horizontal location of the pupil of the eyes ($D_{L}$,$D_{R}$) that defines the short-range gaze when looking at a face placed at different distances ($F_{X},F_{Y}$) in the case of $F_{Z}=0$: (**a**) right eye gaze implementation; (**b**) left eye gaze implementation.

**Figure 11:**
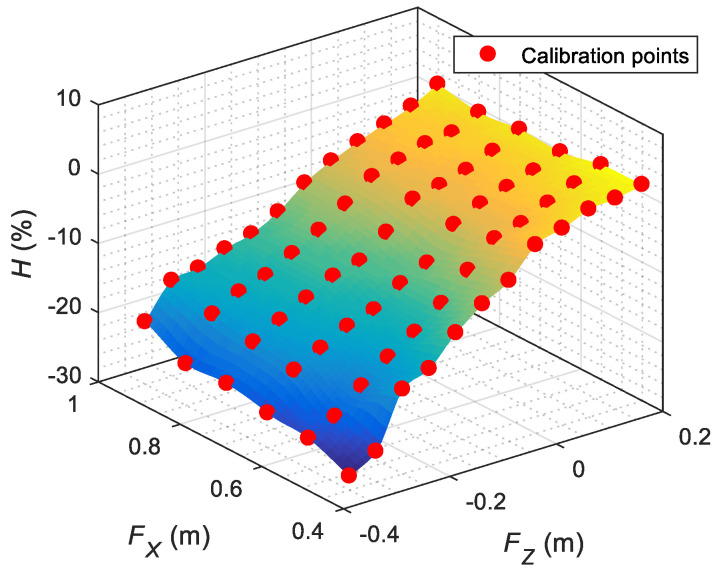
Representation of the vertical location of the pupil of both eyes (H) that defines the short-range gaze when looking at a face placed at different distances ($F_{X},F_{Z}$) in the case of a face centered in front of the mobile robot ($F_{Y}=0$).

Figure 10a,b represent the calibration and interpolation data of the horizontal gaze of the left eye ($D_{L}$) and right eye ($D_{R}$) for ($F_{X},F_{Y})$, with $F_{Z}=0$, assuming a symmetric gaze behavior. The true calibration points are represented with a red circle, and the intermediate points have been obtained using linear interpolation. The calibration data show an abrupt gaze transition originating from when the pupil changes from looking to a face located on its right to a face located on its left. Finally, Figure 11 represents the calibration data of the vertical gaze of both eyes (H) for ($F_{X},F_{Z})$, with $F_{Y}=0$. Again, the calibration points are represented with a red circle, and the intermediate points have been obtained using linear interpolation. The information shown in Figure 10 and Figure 11 defines the short-distance look-at-face gaze from the iconic face.

#### 4.2.2. Determination of a Long-Distance Look-at-Face Gaze from the Iconic Face

The alternative proposed to improve the perception of a long-distance look-at-face gaze action (x > 0.95 m) is based on the calibration of $D_{L}$, $D_{R}$ and H for fixation points placed at different angular orientations (φ, θ). The perception of a long-distance gaze is less precise; thus, the calibration can be limited to changing the horizontal and vertical angular orientation of the fixation point (φ and θ angles) at a fixed distance.

**Figure 12:**
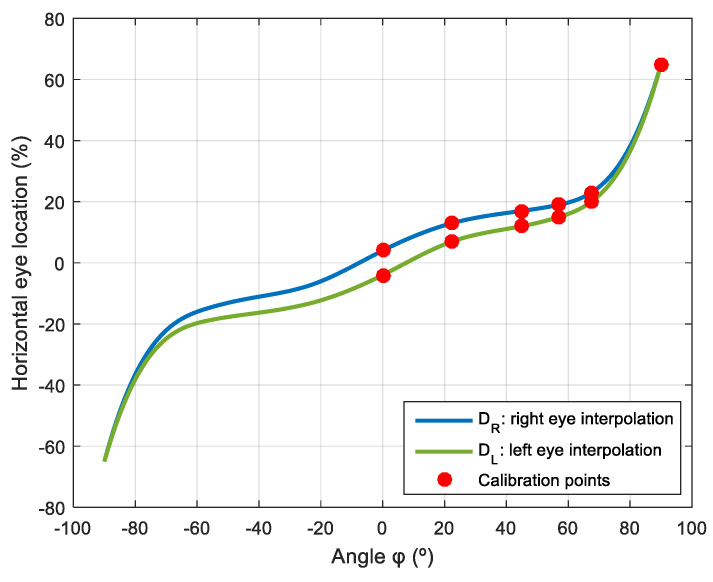
Spline interpolated representation of the horizontal location of the pupil of the left and right eyes ($D_{L}$ and $D_{R}$) that defines the long-range gaze when looking at a face placed at different horizontal angular orientations (φ) in the case of $F_{Z}=0$ and $F_{\varphi }=2.0$ m.

**Figure 13:**
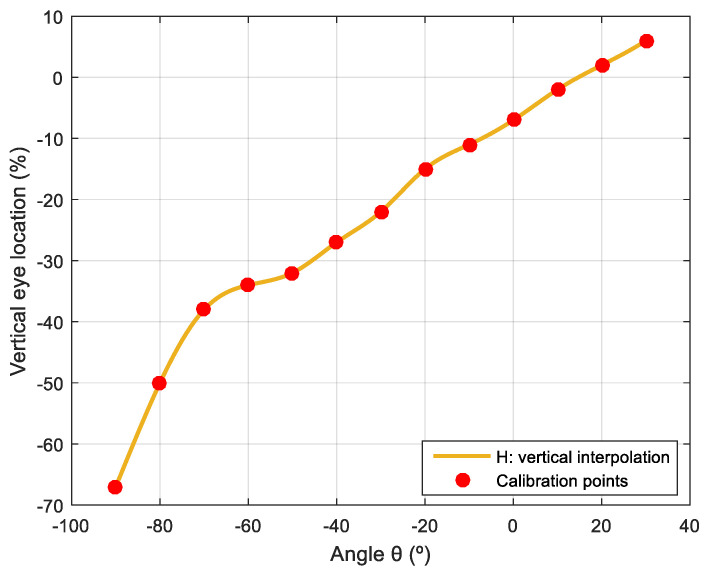
Spline interpolated representation of the vertical location of the pupil of both eyes (H) that defines the long-range gaze when looking at a face placed at different vertical angular orientations (θ) in the case of $F_{Y}=0$ and $F_{\varphi }=2.0$ m.

Table A4 shows the long-range calibration data of $D_{L}$ and $D_{R}$ for different horizontal angular orientations φ with $F_{\varphi }=2.0$ m and $F_{Z}=0$ in order to avoid the influence of vertical deviation in the look-at-face gaze, and Figure 12 shows the spline interpolation performed to obtain the intermediate values. Table A5 shows the long-range calibration data of H for different vertical angular orientations θ in the case of a face centered in front of the mobile robot with $F_{\varphi }=2.0$ m and $F_{Y}=0$ in order to avoid the influence of lateral deviation in the look-at-face gaze, and Figure 13 shows the spline interpolation performed to obtain the intermediate values.

### 4.3. Simulating Saccades during Eye Gaze Contact

This paper proposes the imitation of a cyclic fixation behavior [44] in the gaze of the iconic face when the robot looks at the face of a person placed in front of it. This cyclic fixation sequence is implemented with saccades (rapid, ballistic eye movements that shift gaze between fixation points [39,40]) and must provide a dynamic and familiar gaze sensation [61] that can contribute to enhance the sense of attention from the assistance mobile robot.

**Figure 14:**
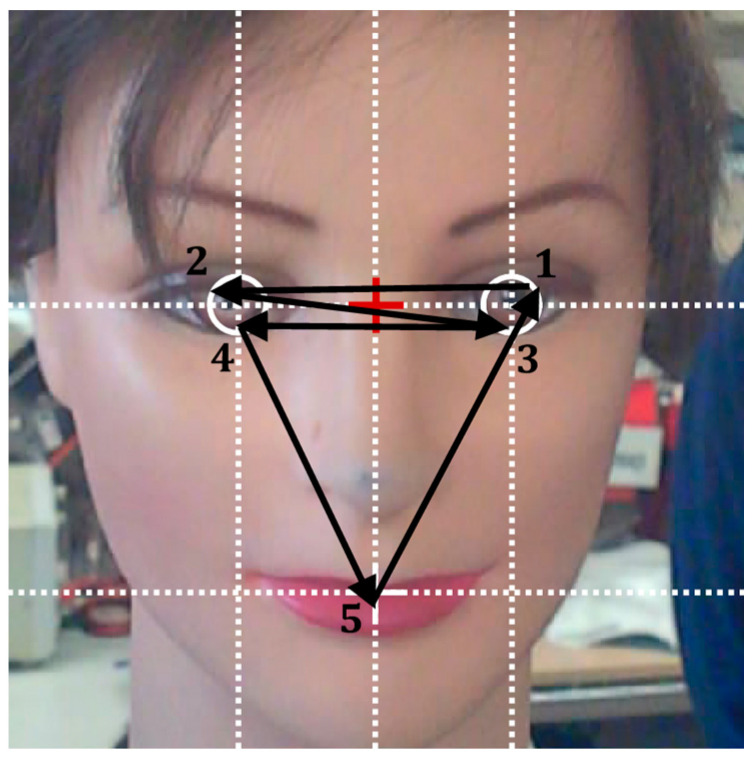
Representation of the saccade trajectories based on the location of the face (red cross) and the fixation points of the left and right eyes and mouth deduced from the face area detected by the Viola–Jones algorithm [54]. The circular saccade sequence represented is 1-2-3-4-5, and the basic fixation time interval is 400 ms.

Figure 14 represents the basic cyclic sequence of saccades proposed to simulate the behavior when looking at a face, which shifts from: left eye (1), to right eye (2), left eye (3), right eye (4) and mouth (5) with a fixation time of 400 ms. The size and location of the face-area detected by the Viola—Jones [54] algorithm in the images of the frontal cameras and the application of the holistic location procedure proposed are used to locate the eyes and mouth of the user.

## 5. Experimental Validation of the Gaze of the Robot

**Figure 15:**
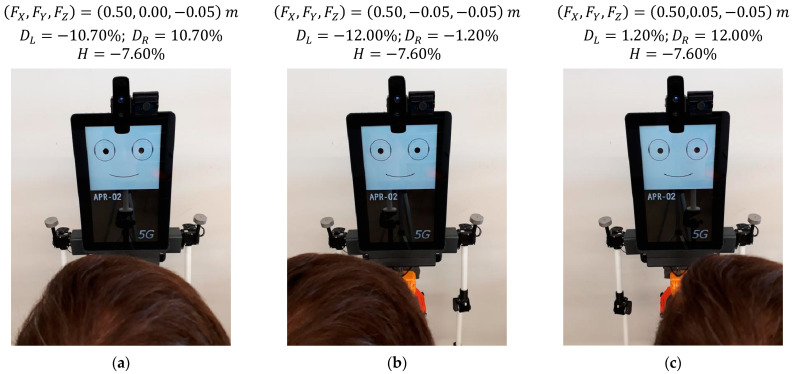
Gaze of the robot following a face performing a lateral displacement: (**a**) face at $F_{Y}$ = 0.00 m; (**b**) face at $F_{Y}$ = −0.05 m; (**c**) face at $F_{Y}$ = 0.05 m.

**Figure 16:**
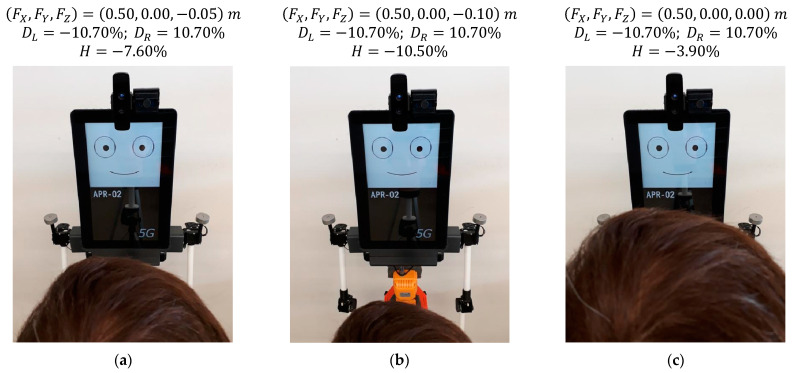
Gaze of the robot following a face at different heights: (**a**) face at $s_{H}$ = 1.55 m; (**b**) face at $s_{H}$ = 1.50 m; (**c**) face at $s_{H}$ = 1.60 m.

**Figure 17:**
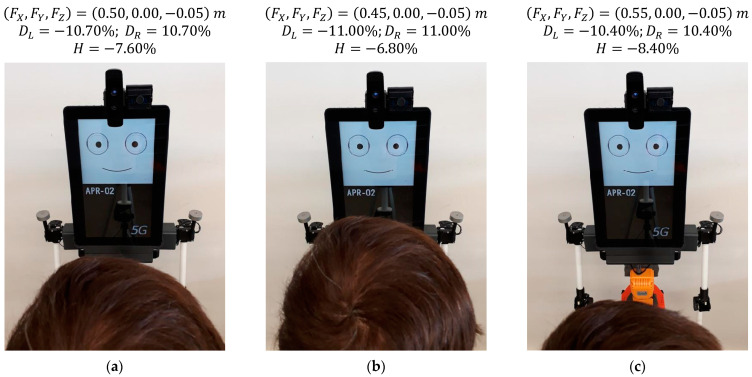
Gaze of the robot looking at a face at different distances: (**a**) face at $F_{X}$ = 0.50 m; (**b**) face at $F_{X}$ = 0.45 m; (**c**) face at $F_{X}$ = 0.55 m.

**Figure 18:**
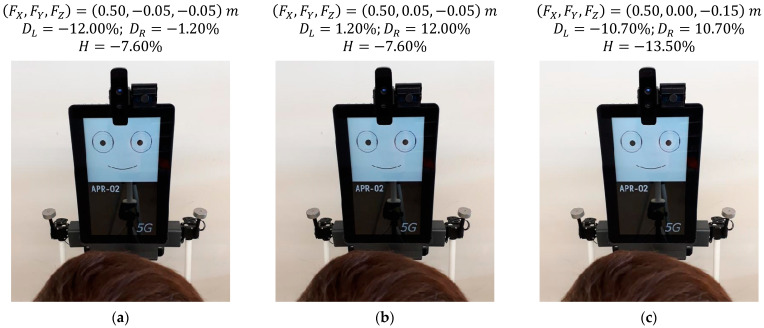
Gaze of the robot at different stages of the cyclic fixation behavior when looking at a face: (**a**) on the left eye of the user; (**b**) on the right eye of the user; (**c**) on the mouth of the user.

**Figure 19:**
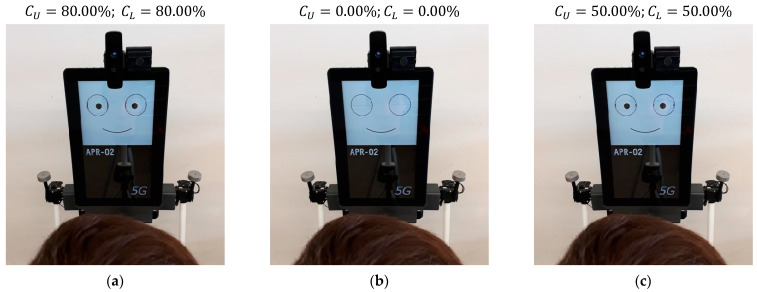
Example of blinking: (**a**) normal gaze; (**b**) closed eyes; (**c**) half-closed eyes.

**Figure 20:**
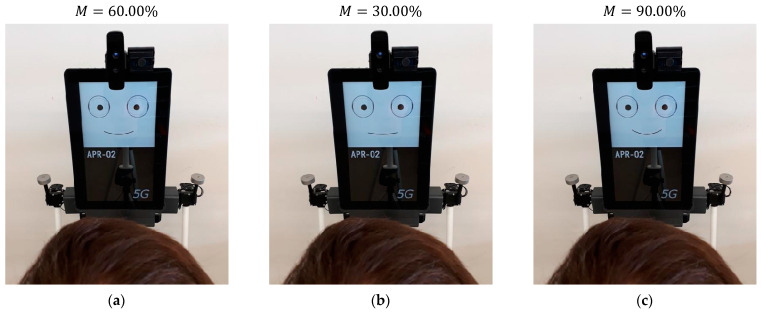
Example of the mouth variations: (**a**) neutral mouth expression; (**b**) attention variation; (**c**) smiling variation.

This section summarizes the work performed to experimentally validate the gaze implemented in the iconic face of the APR-02 mobile robot. The sensation of eye-gaze contact and the sense of attention have been validated successfully by five members of our research laboratory: four male and one female. The Section 5.1, Section 5.2, Section 5.3, Section 5.4, Section 5.5 and Section 5.6 and the Figure 15, Figure 16, Figure 17, Figure 18, Figure 19 and Figure 20 are proposed to visually illustrate the implementation of the eye-gaze contact with a mannequin head in front of the robot. Additionally, an extended demonstration of all these combined implementations is provided in the Supplementary Video S1.

The control of the gaze of the robot is based on the detection of the faces of the people in front of the mobile robot, the estimation of the relative position of these faces, and on pointing the gaze to the face of the nearest person in front of the mobile robot. As described in Section 3.3, the APR-02 mobile robot has an RGB-D and a panoramic RGB camera placed above the screen of the robot that are used to detect the faces of the people in front of the robot by using the Viola–Jones algorithm [54]. The distance to the nearest face in front of the mobile robot is estimated from the depth information provided by the RGB-D camera, and the gaze is automatically focused in the nearest frontal face detected. The position of the pupils of the eyes that defines the gaze is based on the empirical calibrations described in Section 4.2.1 and Section 4.2.2, and the saccades are automatically implemented when the gaze is focused on a face. This automatic implementation simulates human gaze features such as version and vergence if the person in front of the robot moves laterally or moves closer or farther away.

Additionally, following the conclusions of Velichkovsky et al. [22], the gaze is complemented with blinks and small movements of the mouth. The objective of all these combined animations is to avoid the Uncanny Valley effect [62] and assign cognitive and emotional properties to the APR-02 mobile robot in order to enhance the sense of attention from the robot.

### 5.1. Effect of Changing the Horizontal Location of a Face in Front of the Robot

Figure 15 shows the gaze of the robot following a face that is changing its horizontal location in front of the robot (version gaze). The distance from the mannequin to the robot is 0.50 m, the absolute height of the eyes ($s_{H}$) of the mannequin is 1.55 m, and the height of the eyes of the robot ($r_{H}$) is 1.60 m; thus, the eyes are slightly pointing down (H=−7.60%). Figure 15a shows the gaze looking at a person centered in front of the mobile robot, Figure 15b shows the person moved 0.05 m to the right of the robot, and Figure 15c shows the person moved 0.05 m to the left of the robot. The images of Figure 15 show small gaze variations in response to a total lateral displacement of the face of the mannequin of 0.10 m. As an example, in Figure 15, the relative horizontal position of the left pupil $D_{L}$ slightly changes from −10.70% to −12.00% when the gaze of the left eye follows a face from the center to the right. Alternatively, this gaze changes from −10.70% to 1.20% when following a face from the center to the left. These subtle gaze changes are barely perceived in the images but are clearly perceived by a person in front of the mobile robot.

### 5.2. Effect of Changing the Vertical Position of a Face in Front of the Robot

Figure 16 shows the gaze of the robot following a face that changes its vertical position in front of the robot. The distance from the mannequin to the robot is 0.50 m, there is no lateral displacement, and the heights of the eyes ($s_{H}$) are 1.55 m (Figure 16a), 1.50 m (Figure 16b) and 1.60 m (Figure 16c). The images of Figure 16 show small gaze variations in response to a total vertical displacement of the face of the mannequin of 0.10 m. Figure 16 shows that the relative vertical position of the pupil H slightly changes from −7.60% to −10.50% when the gaze of the eyes follows a face that goes down. Alternatively, this vertical position changes from −7.60 to −3.90% when following a face that is going up. Again, these subtle gaze changes are barely perceived in the images but are clearly perceived by a person in front of the mobile robot.

### 5.3. Effect of Changing the Distance of a Face in Front of the Robot

Figure 17 shows the gaze of the robot following a face that changes its distance in front of the robot (vergence gaze). The distances from the mannequin to the robot are 0.50 m (Figure 17a), 0.45 m (Figure 17b) and 0.55 m (Figure 17c). The images of Figure 17 show small gaze variations in response to a total change in the distance of the face of the mannequin of 0.10 m. Figure 17 shows that the relative horizontal positions of the pupils $D_{L}$ and $D_{R}$ slightly change from |10.70%| to |11.00%| when the face approaches and from |10.70%| to |10.40%| when the face moves away. These subtle gaze changes are barely perceived in the images and are barely perceived by a person attentive to the gaze of the robot.

### 5.4. Effect of Saccades in the Gaze of the Robot

Figure 18 shows three stages of the cyclic fixation behavior proposed to imitate the effect of saccades when looking at a face in front of the robot. The mannequin is centered in front of the robot at a distance of 0.50 m, and the height of the eyes ($s_{H}$) of the mannequin is 1.55 m. Figure 18a shows the gaze of the robot looking at the left eye of the face as a fixation point, Figure 18b shows the robot looking at the right eye as a fixation point, and Figure 18c shows the robot looking at the mouth as a fixation point. In this current implementation, the number of eye shifts can vary randomly from 2 to 4 and the fixation time from 400 to 600 ms in order to avoid the generation of fixed predictable cyclic sequences and intervals. The images of Figure 18 show slight gaze variations during this cyclic fixation sequence, which are best perceived when they are implemented as jumps instead of soft transitions or soft displacements. As an example, Figure 18 shows that the relative horizontal position of the pupil of the left eye $D_{L}$ changes from −12.0% to 1.2% when the fixation point shifts from the left eye to the right eye of the mannequin. Similarly, the relative vertical position of the pupil H changes from −7.6% to −13.5% when the fixation point shifts from the right eye to the mouth of the mannequin. Finally, this cyclic fixation behavior imitating an exploration of a face provides a dynamic effect, which is perceived as familiar and natural during an interaction, enhancing the sense of attention and increasing the affinity with the mobile robot.

### 5.5. Effect of Blinking in the Gaze of the Robot

Figure 19 shows the effect of blinking and eyelid control. A blink hides the pupil of the eyes; thus, it has a great effect on the perception of the face. By default, the blink is automatically performed every 1.5 s, as it is perceived as a natural eye reflex. Figure 19a shows the eyelids at their normal position, both covering 20% of the eyes (apertures $C_{U}=80\%$ and $C_{L}=80\%$). Figure 19b shows the eyelids totally closed during a blink, both covering 100% of the eyes (apertures $C_{U}=0\%$ and $C_{L}=0\%$). Figure 19c shows the eyelids half-closed, both covering 50% of the eyes (apertures $C_{U}=50\%$ and $C_{L}=50\%$) as a way to dynamically enhance the gaze of the robot. The implementation of blinks in the gaze of the robot is perceived as familiar and natural during an interaction. The best sense of attention is achieved when eye-blinks are performed as jumps, without smooth transitions. The color of the eyelid and of all the graphic elements of the face can be freely configured, but the use of colors in the face of a mobile robot is a characteristic that requires further analysis by specialized researchers. For example, the image of the iconic face shown in Figure 3 has been configured with gray eyelids to improve the identification of the different parts of the face.

### 5.6. Effect of Subtle Mouth Animations

The implementation of subtle animations in the mouth was initially unplanned, but it is the natural remaining step after the implementation of saccades and blinking in the gaze in order to enhance the sense of attention from the robot.

The animation of the mouth is synchronized (or implemented) with the saccades. The basic parameter that modifies the amplitude of the smile M is randomly increased up to 50% of its fixed value. The objective of the subtle random variation of the amplitude of the smile is to provide a dynamic perception and to enhance the sense of attention from the mobile robot. Figure 20a shows the mouth used to express a positive-neutral facial expression in the robot, with M=60.00%, selected in [47], to encourage interaction. Figure 20b shows a lower smiling degree achieved with M=30.00%, and Figure 20c shows a higher smiling degree achieved with M=90.00%. Finally, these subtle mouth changes are perceived in the images but are not directly perceived by a person attentive to the gaze of the robot, since they are similar to the subtle micro-emotions expressed by the human smile [63]. In this case, the best sense of attention is also achieved when the mouth movements are performed as jumps, without smooth transitions.

## 6. Discussion and Conclusions

This paper proposes enhancing the sense of attention from an assistance mobile robot prototype by improving eye-gaze contact from its iconic face displayed on a flat screen. This iconic face was implemented with big round eyes and a mouth depicted with a single line. The inclusion of this face was considered a determining factor to develop assistance services, and the gaze and emotion displayed in the face were treated as other actuators of the robot. The implementation of eye-gaze contact from the iconic face is a problem because of the difficulty of simulating real 3D spherical eyes in a 2D image considering the perspective of the person interacting with the mobile robot.

In general, the gaze in a robot is implemented in order to provide the basic impression of a responsive robot [19,25]. The gaze implemented originally in the assistance mobile robot used in this paper had seven predefined gaze orientations: forward, up, down, half left, half right, left, and right, in all cases with parallel eyes fixed on infinity. In this case, the use of a deterministic computation of the angular orientation of the spherical eyes was not convincing because the eyes of this iconic face were perceived as 2D objects, and the geometric projection of 3D spherical eyes did not provide a convincing eye-gaze effect. The method implemented in this paper to maximize the perception of eye-gaze contact from the face of the robot is based on a manual calibration of the location of the pupils relative to the distance and orientation of the face of the user interacting with the robot. The method implemented provides a total of 169 eye-gaze calibration points and interpolation recommendations. Two basic eye-gaze calibration procedures have been implemented. A detailed short-distance eye-gaze calibration enables an accurate imitation of the looking-at-face gaze in the case of a user placed in front of the mobile robot, while a long-distance eye-gaze calibration enables a rough imitation of the look-at-face gaze in the case of a user away from the robot. The difference between these two calibrations is that in the short-distance calibration, the user interacting with the robot must accurately and precisely perceive eye-gaze contact from the robot, while in the long-distance calibration, the user perception is less precise. The implementation of this robotic gaze has been validated with five people who work regularly with robots. The limitation of this method proposed to maximize the perception of eye-gaze contact is that it has been optimized for the eye dimensions implemented in the iconic face used in the assistant mobile robot APR-02. The general application of this methodology remains an open question that will require the development of further analyses with other robotic face designs, for example, evaluating the use of the pupillary distance as a reference to normalize the calibration data provided.

The direct use of calibration data as a strategy to improve eye-gaze contact from the face of the robot has provided an optimal gaze in a short-range interaction and the best perception that the robot is attentive to the user. Further enhancements regarding sense of attention have been achieved with the implementation of a cyclic face exploration sequence based on the holistic location of the eyes and mouth in the image of the user placed in front of the robot. This cyclic face exploration is implemented with saccades, using a deterministic eye-gaze sequence shifting from the left to the right eye several times and then shifting to the mouth and starting again. This exploration sequence can be adapted depending on the cultural background of the user interacting with the robot or depending on the objective of the eye-gaze contact [44].

The practical application of this responsive gaze in the assistant mobile robot APR-02 is based on the information provided by two frontal onboard cameras and on the application of the Viola–Jones face detection algorithm [54]. The use of a face detection algorithm provides a valuable indication of the existence of a person looking at or oriented to the mobile robot. In this case, the frontal depth camera also provides an estimate of the distance of the faces detected in a short distance range in front of the mobile robot for precise gaze control, while the location of the faces of the most distant people is roughly estimated from the information gathered by the onboard LIDAR.

Finally, the sense of attention has been maximized by simulating eye-blinks and small mouth movements. The best sense of attention has been achieved when the saccades, eye-blinks and mouth movements have been performed as jumps, without smooth transitions. The familiar human-like behavior achieved with the combination of all these dynamic face effects has contributed to the assignation of cognitive and emotional properties to an assistance mobile robot prototype displaying an iconic face in a flat screen and has improved the affinity with the robot. The development of this perception agrees with Yoshikawa et al. [3], who concluded that a responsive gaze provides a strong feeling of being looked at, with Fukayama et al. [16], who concluded that there is a correlation between user impression and the gaze of a robot, with Velichkovsky et al. [22], who also concluded that the simulation of a human gaze can provoke the assignation of cognitive and emotional properties to a robot, and with Mori et al. [62], who proposed the Uncanny Valley effect to model the affinity with a human-like robot, suggesting that the worst affinity is obtained in the case of a static robot.

The complete procedure proposed in this paper to improve the sense of attention can be applied to robots with mechatronic faces, although then the limitation will be the continuous mechanical implementation of instantaneous saccades, eye-blinks and small mouth movements. Alternatively, the implementation of a precise eye-gaze contact may also have promising applications in virtual reality [64] and in future applications of augmented reality [65].

As a future work, the implementation of eye-gaze contact from the robot will include an estimation of the gaze of the user in front of the robot [66,67] in order to evaluate the implementation of new mutual-gaze features such as sharing the focus of attention or redirecting the focus of attention. Additionally, the expressivity of the mobile robot will be implemented as a specific agent combining gaze control, face control and arms control in order to adequately imitate human behaviors in complex humanoid robots.

## Figures and Tables

**Table 1:** Summary of the parameters defined in the eye model represented in Figure 1.

Parameter Symbol	Definition
X,Y,Z	Coordinate system defined by the center of the eyes
F	Fixation point
$F_{X},F_{Y},F_{Z}$	Coordinates of the fixation point
Plane DY	Sight plane
D, Y	Coordinates of the sight plane: Plane DY
$F_{D};F_{Y};F_{\varphi }$	Coordinates and distance of the fixation point in the sight plane
$p_{D}$	Pupillary distance
$e_{Ø}$	Diameter of the eyeball
θ	Sight angle referred to the XY plane
φ	Angle of the fixation point referred to the coordinates D, Y
$\varphi_{L},\varphi_{R}$	Angular deviation of the left and right eyes to the fixation point.

**Table 2:** Summary of the parameters of the iconic face of the assistance mobile robot represented in Figure 3.

Parameter	Definition	Size (px)	Size (mm)	Relationships
$s_{H}$	Total height of the visible area of the screen	1920	293.76	
$s_{W}$	Total width of the visible area of the screen	1080	165.24	
$f_{H}$	Face height	960	146.88	
$f_{W}$	Face width	1080	165.24	$f_{W}/f_{H}$ = 112.50%
$e_{H}$	Eyes height	341	52.17	$e_{H}/f_{H}$ = 35.52%
$m_{H}$	Mouth height	778	119.03	$m_{H}/f_{H}$ = 81.04%
$p_{D}$	Pupillary distance	555	84.92	$p_{D}/f_{H}$ = 57.81%
$e_{Ø}$	Eye diameter	317	48.50	$e_{Ø}/f_{H}$ = 33.02%
$p_{Ø}$	Pupil diameter	86	13.16	$p_{Ø}/f_{H}$ = 8.96%
$s_{Ø}$	Sclera diameter	286	43.76	
$l_{U}$	Default upper eyelid	30	4.59	
$l_{L}$	Default lower eyelid	30	4.59	
$r_{W}$	Rim width	8	1.22	
$p_{R}$	Right pupil horizontal displacement	0	0	
$p_{L}$	Left pupil horizontal displacement	0	0	
$p_{H}$	Pupils vertical displacement	0	0	

## Data Availability

Calibration data used in this paper is provided in Appendix A.

## Supplementary Materials

The following supporting information can be downloaded at: https://www.mdpi.com/article/10.3390/s22114282/s1, Video S1: Short demonstrative video of the eye-gaze contact effect.

## Appendix A

**Table A1:** Value of $D_{L}$ (relative horizontal deviation of the pupil of the left eye) depending on the location of the fixation point ($F_{X},F_{Y}$) in the case of $F_{Z}=0$. Short-range horizontal calibration data for $F_{X}\;\leq 0.95\;m$ and $F_{Y}\;\leq 0.40\;m$.

$F_{Y}\;\left(m\right)\;$	$F_{X}\;\left(m\right)$					
	0.45	0.55	0.65	0.75	0.85	0.95
**0**	−11.0%	−10.4%	−7.6%	−7.0%	−6.0%	−6.0%
**0.05**	2.0%	0.4%	0.0%	2.0%	0.4%	−0.8%
**0.10**	6.6%	3.2%	1.2%	3.8%	2.0%	3.4%
**0.20**	7.2%	4.8%	3.4%	5.4%	4.0%	2.6%
**0.30**	9.0%	6.6%	6.0%	5.6%	2.2%	1.6%
**0.40**	11.6%	3.6%	6.0%	5.4%	5.4%	1.8%

**Table A2:** Value of $D_{R}$ (relative horizontal deviation of the pupil of the right eye) depending on the location of the fixation point ($F_{X},F_{Y}$) in the case of $F_{Z}=0$. Short-range horizontal calibration data for $F_{X}\leq 0.95\;m$ and $F_{Y}\leq 0.40\;m$.

$F_{Y}\;\left(m\right)\;$	$F_{X}\;\left(m\right)$					
	0.45	0.55	0.65	0.75	0.85	0.95
**0**	11.0%	10.4%	7.6%	7.0%	6.0%	6.0%
**0.05**	12.2%	11.8%	11.8%	11.4%	12.4%	13.0%
**0.10**	15.0%	12.6%	13.2%	11.2%	13.2%	12.6%
**0.20**	17.0%	10.0%	14.8%	12.2%	14.4%	13.8%
**0.30**	18.8%	14.0%	15.4%	12.4%	16.4%	14.0%
**0.40**	20.8%	14.6%	17.4%	12.6%	13.4%	15.0%

**Table A3:** Value of H (relative vertical deviation of the pupil of both eyes) depending on the location of the fixation point ($F_{X},F_{Z}$) in the case of $F_{Y}=0$. Short-range vertical calibration data obtained for $F_{X}\leq 0.95\;m$ and $-0.35\;m\leq F_{Z}\leq 0.20\;m$.

$F_{Z}\;\left(m\right);\;s_{H}\;\left(m\right)\;$	$F_{X}\;\left(m\right)$					
	0.45	0.55	0.65	0.75	0.85	0.95
**−0.35; 1.25**	−28%	−25.6%	−25%	−23.8%	−24%	−21%
**−0.30; 1.30**	−25.6%	−23.6%	−20.0%	−19.0%	−18.0%	−16.2%
**−0.25; 1.35**	−17.8%	−20.4%	−18.0%	−17.0%	−16.0%	−15.6%
**−0.20; 1.40**	−16.0%	−17.4%	−16.0%	−15.0%	−14.8%	−14.0%
**−0.15; 1.45**	−12.0%	−15.0%	−14.8%	−13.0%	−12.8%	−13.0%
**−0.10; 1.50**	−9.0%	−12.0%	−12.2%	−12.0%	−10.6%	−11.0%
**−0.05; 1.55**	−6.8%	−8.4%	−10.4%	−9.0%	−8.0%	−8.0%
**0; 1.60**	−2.8%	−5.0%	−6.0%	−5.4%	−5.2%	−6.0%
**0.05; 1.65**	−1.6%	−4.0%	−4.2%	−4.6%	−4.0%	−4.4%
**0.10; 1.70**	0.0%	−2.2%	−3.0%	−3.6%	−2.8%	−3.0%
**0.15; 1.75**	0.4%	−0.6%	−1.8%	−2.0%	−2.4%	−1.6%
**0.20; 1.80**	1.2%	1.0%	−0.2%	0.0%	−0.6%	0.4%

**Table A4:** Values of $D_{L}$ and $D_{R}$ depending on the horizontal angular orientation of the fixation point (φ) at $F_{\varphi }=2.0$ m. Long-range horizontal calibration data obtained for the left and right eyes.

	φ					
	0°	22.5°	45.5°	57.0°	67.5°	90.0°
$D_{L}$	−4%	7%	12%	15%	20%	65%
$D_{R}$	4%	13%	17%	19%	23%	65%

**Table A5:** Value of H depending on the vertical angular orientation of the fixation point (θ) at $F_{\varphi }=2.0$ m. Long-range eye-gaze calibration data obtained for both eyes.

	θ												
	30°	20°	10°	0°	−10°	−20°	−30°	−40°	−50°	−60°	−70°	−80°	−90°
H	6%	2%	−2%	−7%	−11%	−15%	−22%	−27%	−32%	−34%	−38%	−50%	−67%
